# Molecular mechanisms of growth depression in broiler chickens (*Gallus Gallus domesticus*) mediated by immune stress: a hepatic proteome study

**DOI:** 10.1186/s40104-021-00591-1

**Published:** 2021-07-13

**Authors:** Aijuan Zheng, Anrong Zhang, Zhimin Chen, Shoaib Ahmed Pirzado, Wenhuan Chang, Huiyi Cai, Wayne L. Bryden, Guohua Liu

**Affiliations:** 1 Key Laboratory of Feed Biotechnology of Ministry of Agriculture and Rural Affairs, Institute of Feed Research, Chinese Academy of Agricultural Sciences, No.12 Zhongguancun south street, Haidian district, Beijing, 100081 China; 2 School of Agriculture and Food Sciences, University of Queensland, Gatton, QLD 4343 Australia

**Keywords:** Broiler chickens, Hepatic proteome, Immune stress, Lipopolysaccharide

## Abstract

**Background:**

Immunological stress decreases feed intake, suppresses growth and induces economic losses. However, the underlying molecular mechanism remains unclear. Label-free liquid chromatography and mass spectrometry (LC-MS) proteomics techniques were employed to investigate effects of immune stress on the hepatic proteome changes of Arbor Acres broilers (*Gallus Gallus domesticus*) challenged with *Escherichia coli* lipopolysaccharide (LPS).

**Results:**

Proteomic analysis indicated that 111 proteins were differentially expressed in the liver of broiler chickens from the immune stress group. Of these, 28 proteins were down-regulated, and 83 proteins were up-regulated in the immune stress group. Enrichment analysis showed that immune stress upregulated the expression of hepatic proteins involved in defense function, amino acid catabolism, ion transport, wound healing, and hormone secretion. Furthermore, immune stress increased valine, leucine and isoleucine degradation pathways.

**Conclusion:**

The data suggests that growth depression of broiler chickens induced by immune stress is triggered by hepatic proteome alterations, and provides a new insight into the mechanism by which immune challenge impairs poultry production.

## Background

Intensive poultry production is conducted in an environment that imposes many stressors on the bird. The stressed bird instigates an integrated response to maintain homeostasis through cross-talk between the central nervous, endocrine and immune systems [1]. Stressors in the bird’s environment, include feeding management, overcrowding, temperature extremes, dust and litter condition, pathogen challenges, vaccination, and psychological factors [2, 3]. All of these stressors can have a cumulative impact on poultry behavior and physiology, thus affecting the immune response and inducing immunologically mediated stress or immune stress [4, 5]. Immune stress is harmful to the bird and can be mitigated by improving the bird’s environment. Studies have shown that stress dysregulates the immune response by increasing the release of inflammatory cytokines and stress hormones [6, 7], reducing NK cell activity, lymphocyte populations, lymphocyte proliferation, antibody production and reactivating latent viral infections [8, 9]. In response to an immune challenge, the appetite and growth performance of the bird will decline [10–12]. Immune stress can also disrupt the balance and composition of the cecal microflora, impair intestinal mucosal immune function, and reduce ileal protein digestibility [13].

A bird or animal’s metabolic priorities are rearranged in response to immune stress, resulting in the redistribution of nutrients away from muscle protein deposition and growth to support upregulation of the immune response [14, 15]. The liver plays a pivotal role in nutrient metabolism, and nutrient repartitioning following an immune challenge when it is enriched with components of the immune system, including macrophages and natural killer T-cells; highlighting the vital role of the liver in immunology [16].

Despite extensive research on the effects of immune stress in broiler chickens, changes in the avian hepatic proteome and the molecular mechanisms induced by an immune insult are not well understood. Proteins as the functional carrier of genes can provide both genomic and functional information [17]. Proteomics represents a new strategy to delineate the molecular basis of the physiological changes in the liver during chicken growth [18, 19]. This approach determines the differential patterns of protein abundance and has been used to demonstrate their functional relationships to external factors [20, 21]. Lipopolysaccharide (LPS) injection is a classical model for inducing immune stress in broiler chickens [7, 10, 22, 23]. In the present experiment, this model was used to investigate the hypothesis that changes in the expression of the hepatic proteome of broilers occur following immune challenge and help explain the response of the bird. Our findings clarify protein expression and biological process changes in the liver of immunologically challenged broilers and provides further information to assist in maintaining the health and productivity of meat or broiler chickens.

## Methods

### Materials and reagents

All chemicals were purchased from Sigma-Aldrich (St. Louis., Missouri, USA) except modified sequencing grade trypsin that was bought from Promega (Madison, WI, USA). LPS from *E. coli* (O55:B5) was used in the present experiment.

### Bird management

A total of 144 one-day-old, male, Arbor Acres (AA) broiler chickens were purchased from Huadu Chicken Co. (Beijing, China). The chicks were randomly divided into two groups: challenged with saline (control group) or LPS (treatment group). Each group had 6 replicates with 12 birds in each replicate. The distribution of cages was arranged to avoid any location effects within the poultry house. The chickens were reared in two phases and fed a starter diet during d 0–21 and a grower diet during d 22–42. The composition of these corn-soybean based diets are shown in Table 1. All chickens were inoculated and subjected to a photoperiod of 16 h light and 8 h dark in accordance with the AA Broiler Management Guide. The room temperature was maintained at 33–35 °C on d 0–3, at 32–34 °C on d 4–7 and gradually reduced to the maintenance temperature of 20 °C by d 42. The relative humidity was kept at 70% during the first week and thereafter at about 60%.

**Table 1:** Ingredient and nutrient composition of experimental broiler diets

	Starter (1–21 d), g/kg	Grower (22–42 d), g/kg
**Ingredient**		
Corn	593.1	604.2
Soybean meal	298.8	288.7
Cotton seed meal	50.0	30.0
Soybean oil	15.1	39.8
*L*-Lysine	1.5	0.9
*DL*-Methionine	1.4	1.6
Limestone	12.7	10.2
CaHPO_4_	19.4	16.6
NaCl	3.0	3.0
Choline chloride	2.0	2.0
Vitamin premix	0.3	0.3
Mineral premix^a^	1.0	1.0
Zeolite powder	1.7	1.7
Total	1000	1000
**Nutrient concentrations**^**b**^		
Metabolic energy, MJ/kg	12.35	13.02
Crude protein	211.8	198.4
Calcium	10.1	8.5
Available phosphorus	4.5	4.0
Total phosphorus	6.9	6.3
Lysine	11.4	10.5
Methionine	4.9	4.8
Methionine + Cysteine	8.3	8.1
Threonine	7.7	2.2

^a^The premix provided the following per kg diet: vitamin A 10, 000 IU, vitamin D_3_ 2,000 IU, vitamin E 10 IU, vitamin K_3_ 2.5 mg, vitamin B_1_ 1 mg, vitamin B_2_ 6 mg, vitamin B_3_ 10 mg, vitamin B_5_ 40 mg, vitamin B_6_ 3 mg, vitamin B_11_ 0.3 mg, vitamin B_12_ 0.01 mg, biotin 0.12 mg, Cu (as copper sulfate) 8 mg, Fe (as ferrous sulfate) 80 mg, Mn (as manganese sulfate) 60 mg, Zn (as zinc sulfate) 40 mg, Se (as sodium selenite) 0.15 mg, I (as potassium iodide) 0.35 mg

^b^Calculated values

### Experimental treatments and LPS administration

For the first 5 weeks of the study all birds were maintained in a similar manner. On d 36, 38, and 40, all chickens (each group had 6 replicates with 12 birds in each replicate) were injected intravenously with either 1 mL sterile saline (control group) or LPS (treatment group) dissolved in saline at an approximate dose of 5.0 mg/kg body weight (LPS or immune stress group). The injection protocol is the established method used when inducing an immunological challenge with LPS [4, 7, 10, 22, 23]. The protocol commenced at 5 weeks of age to avoid endocrine and physiological changes that occur during the starter phase and to permit additional muscle samples to be collected for meat analysis; results reported separately.

#### Performance parameters

The body weight of all birds in each replicate was measured on d 36 (before the first injection, W_0_), d 38 (2 days after the first injection, W_38_), d 40 (2 days after the second injection, W_40_) and d 42 (2 days after the third injection, W_42_). The change in body weight caused by saline or LPS treatment was expressed as body weight gain (W_1_ = W_38_- W_0_, W_2_ = W_40_- W_0_, W_3_ = W_42_- W_0_). W_1–3_ indicates body weight gain after the first, second or third injection of LPS. Mortality was recorded daily.

### Sample collection and parameters determined in blood

On d 42, all birds were weighed after a 12 h-fast. Three birds from each replicate were selected randomly, electrically stunned, and manually slaughtered within 5 min [24]. Blood was collected using vacutainer tubes. The serum, obtained by centrifugation at 1,500 × *g* for 15 min, was used for the determination of hormones and inflammatory factors. The concentrations of adrenocorticotropic hormone (ACTH), corticosterone (CORT), growth hormone (GH) and insulin-like growth factor-1 (IGF-1), interleukin-1β (IL-1β), interleukin-6 (IL-6), and tumour necrosis factor-α (TNF-α) were determined by quantitative sandwich enzyme immunoassay using commercial kits (Beijing North Institute of Biological Technology, Beijing, China), according to the manufacturer’s instructions.

The middle section of the major or right lobe of the liver was sampled and washed with PBS buffer (NaCl 8 g/L, Na_2_HPO_4_ 1.44 g/L, KH_2_PO_4_ 0.24 g/L, KCl 0.2 g/L, pH 7.2) to remove any blood and contaminants on the surface. A liver sample (about 2 g) was taken and put into 5 mL ultra-low temperature freezing tubes (Free Sterile). Samples were immediately frozen in liquid nitrogen and stored at − 80 °C. Likewise, intestinal and muscle samples were also collected and the outcome of their analyses will be published elsewhere.

### Protein extraction and digestion

The liver samples of three chickens from each replicate (cage) were combined as a biological replicate, homogenized by pestle in liquid nitrogen. Six biological replicates of each group were analyzed. Protein extraction was performed as previously described [18]. In short, after homogenization the samples were then mixed with a lysis buffer containing 8 mol urea, 2 mol thiourea, 4% 3-[(3-cholamidopropyl) dimethylammonio]-1-propanesulfonate, 20 mmol Trisbase, 30 mmol dithiothreitol (DTT), and protease inhibitors in ice for 30 min. The sample was then centrifuged at 15,000 × *g* for 20 min at 10 °C to remove the insoluble fractions. Three volumes of ice-cold acetone were added to the recovered supernatant and allowed to stand at 20 °C for 4 h to precipitate the proteins. Subsequently, the protein pellets were centrifuged at 8,000 × *g* at 10 °C for 20 min. The supernatant was discarded, followed by extraction of the protein pellet at room temperature. The recovered proteins were re-suspended in 100–150 μL of 5 mol urea, and protein concentration was quantified by the Bradford assay after diluting 50 times. Of each sample, 200 μg of proteins were used by adding four volumes of 40 mmol NH_4_HCO_3_, mixing with DTT (final concentration 10 mmol) for 1 h, and then alkylating with iodoacetamide (final concentration 50 mmol) for 1 h in the dark. The surplus iodoacetamide was quenched by DTT (final concentration 30 mmol). To digest protein into peptides, sequencing grade modified trypsin was used (enzyme/protein ratio of 1:100 (W/W)) at 37 °C for 14 h. The enzymatic digestion was stopped by adding 1 μL of formic acid to the solution. The digested peptide samples were desalted using a C18 column (Agilent Technologies Inc., Santa Clara, CA, USA). The eluted peptide solution was collected and extracted using a SpeedVac system (RVC 2–18, Marin Christ, Osterod, Germany) and stored at −80 °C for subsequent LC-MS/MS analysis.

### Liquid chromatography and mass spectrometry (LC − MS/MS) analysis

The digested peptide samples were re-dissolved in 50 μL of 0.1% formic acid. Three replicates of each sample were run using a Q-Exactive mass spectrometer (Thermo Fisher Scientific, USA) and coupled to the EASY-nLC 1000 system using a nano electrospray ion source (Thermo Fisher Scientific, USA). To enrich the peptide samples, they were first loaded onto a 2 cm long trap column (75 μm inner diameter fused silica containing 3 μm Aqua C18 beads, Thermo Fisher Scientific, USA) for 2 min in buffer A (0.1% acetic acid) at a flow rate of 10 μL/min. Secondly, the peptides were separated by an analytical column (15 cm long, 50 μm inner diameter fused silica column filing with 2 μm Aqua C18 beads, Thermo Fisher Scientific, USA) using a 120 min gradient. Peptides were gradient eluted for 110 min with a linear gradient from 8% to 30% acetonitrile at a flow rate of 300 nL/min. The eluting peptides from the analytical column were directly infused into a Q-Exactive mass spectrometer via electrospray ionization. The settings for a data-dependent mode to collect the MS and MS/MS data were as follows: one full scan (resolution 70,000 at 400 m/z; 350 to 1,600 m/z) followed by top 20 MS/MS scans using higher-energy collisional dissociation in the linear ion trap mass spectrometer (resolution: 15,000, isolation window: 2 m/z, normalized collision energy: 28) using dynamic exclusion (charge exclusion: unassigned 1, > 8; peptide match: preferred; exclude isotopes: on; dynamic exclusion: 30 s). For identification and abundance level quantification of proteins, the MS/MS data in RAW were retrieved using Xcalibur (version 3.0, Thermo Fisher Scientific, USA) and searched using in-house PEAKS software (version 8.5, Bioinformatics Solutions Inc., CAN).

A database containing protein sequences of *Gallus Gallus domesticus* including common contaminants was downloaded from NCBI and used, totaling to 76,213 entries (downloaded 25 June, 2020). The parameters of the search database were as follows: trypsin; maximum missed cleavage: 2; precursor ion and MS/MS tolerances: 15 ppm and 0.05 Da; a fixed modification: carbamidomethyl (C, + 57.02); and a variable modification: methionine oxidation (M, + 15.99), asparagine and glutamine deamination (+ 0.984 Da). The fusion-decoy database search strategy with threshold false discovery rate (FDR ≤ 1%) was used to control the FDR at both the protein and peptide levels. A protein was considered as identified only if it had at least one unique peptide. To quantify the relative protein abundance in the livers of broiler chickens both from the control group and immune stress group, three replications of each sample were performed in the quantification module of PEAKS software (version 8.5) via a label-free strategy. Feature detection was performed separately on each sample using the expectation-maximization algorithm. Using the high-performance retention time alignment algorithms, the features of the same peptide from three replicates of each sample were reliably aligned [25]. Normalization was done by dividing each matrix by a factor of the samples obtained as follows: the total ion current (TIC) of the individual sample / the TIC of the reference sample. Quantification of protein abundance in the livers in all samples of broiler chickens was done using the sum of the three highest ion peak intensities of the tryptic peptides.

### GO term enrichment analysis

To understand the biological implications of the identified proteins in the liver of broiler chickens, identifiers of protein symbol ID numbers were used as an input for GO term enrichment (functional classes and pathway) using ClueGOv2.3.2, a Cytoscape plug-in (http://www.ici.upmc.fr/cluego/) [26]. The number of proteins identified from the samples was compared with the number of functionally GO annotated proteins in the entire broiler chicken (*Gallus Gallus domesticus*) genome for enrichment analysis. The significantly enriched GO terms in biological processes and pathways were reported using a right-sided hyper-geometric test and only a *P*-value < 0.05 was considered. Then, Bonferroni step-down procedure was used to correct the *P*-value to control FDR. Functional grouping of the terms was based on GO hierarchy. The tree level was ranged from 3 to 8, and kappa score level was 0.4. For comparison purpose, sharing 65% of the terms was considered to be merged.

### Protein–protein interaction analysis

A protein–protein interaction network of differential proteins was constructed using the STRING 11.0 (http://string-db.org/) [27]. The network nodes represent proteins, and the edges represent the predicted functional associations.

### Statistical analysis

Means of replicate were used as the experimental unit for statistical analysis. The data of blood parameters were analyzed by Independent-Samples T-Test module using SPSS 17.0 software (version 17.0, SPSS Inc., Chicago, IL, USA). Results are presented as the mean ± SE. Differences between means were considered statistically significant at *P* < 0.05.

Proteins from different samples were considered to be significantly changed in their abundance only when they attained the criteria (*P*-value < 0.05 and a fold change of > 1.5 or < 0.5).

## Results

Growth and wellbeing of all chicks was normal for the first 5 weeks of the study or until the LPS challenge was introduced.

### Effects of body weight gain of broilers challenged with LPS

The effects of immune stress on body weight gain of broilers is shown in Table 2. Body weight gain in broilers injected with LPS was significantly lower than in the unchallenged broilers.

**Table 2:** Body weight gain of broilers challenged with LPS

TREATMENT	W_0_, g	W_1_, g	W_2_, g	W_3_, g
Control group	1966 ± 116	182 ± 9.7^a^	389 ± 22.9^a^	423 ± 27.6^a^
Immune stress group	1966 ± 107	−22.7 ± 26.9^b^	97.9 ± 46.4^b^	112.3 ± 46.7^b^
*P*-value	0.49	0.0001	0.0001	0.0001

*W*_*0*_, Initial body weight before injection of LPS; *W*_*1*_, Body weight gain 2 days after the first injection of LPS; *W*_*2*_, Body weight gain 2 days after the second injection of LPS; *W*_*3*_, Body weight gain 2 days after the third injection of LPS

^a,b^ In the same column, values with the same or no letter superscripts mean no significant difference (*P* > 0.05), while with different letter superscripts mean significant difference (*P* < 0.05)

### Changes of serum hormones and cytokines of broilers challenged with LPS

As shown in Table 3, the serum concentrations of ACTH, CORT, IL-1β, TNF-α and IL-6 in broilers injected with LPS were significantly higher than in the unchallenged broilers. However, GH and IGF- І concentrations in serum decreased significantly in the broilers from the immune stress group.

**Table 3:** The concentrations of serum hormones and cytokines in broilers challenged with LPS

	TNF-α, fmol/mL	IL-1β, pg/mL	IL-6, ng/mL	GH, ng/mL	CORT, pg/mL	ACTH, pg/mL	IGF-І, ng/mL
Control	5.88 ± 0.09^a^	0.087 ± 0.006^a^	60.06 ± 6.87^a^	1.37 ± 0.11^a^	8.36 ± 0.67^a^	5.91 ± 0.63^a^	80.46 ± 4.78^b^
LPS	9.45 ± 0.55^b^	0.223 ± 0.041^b^	83.93 ± 2.30^b^	1.12 ± 0.03^b^	10.26 ± 0.35^b^	8.24 ± 0.83^b^	71.53 ± 3.48^a^
*P*-value	0.000	0.000	0.000	0.000	0.047	0.047	0.030

^a,b^ In the same column, values with the same or no letter superscripts mean no significant difference (*P* > 0.05), while with different letter superscripts mean significant difference (*P* < 0.05)

### Qualitative differential analysis of hepatic proteome in broiler chickens between the control and the immune stress group

#### Protein numbers expressed in the liver of broiler chickens

**Fig. 1:**
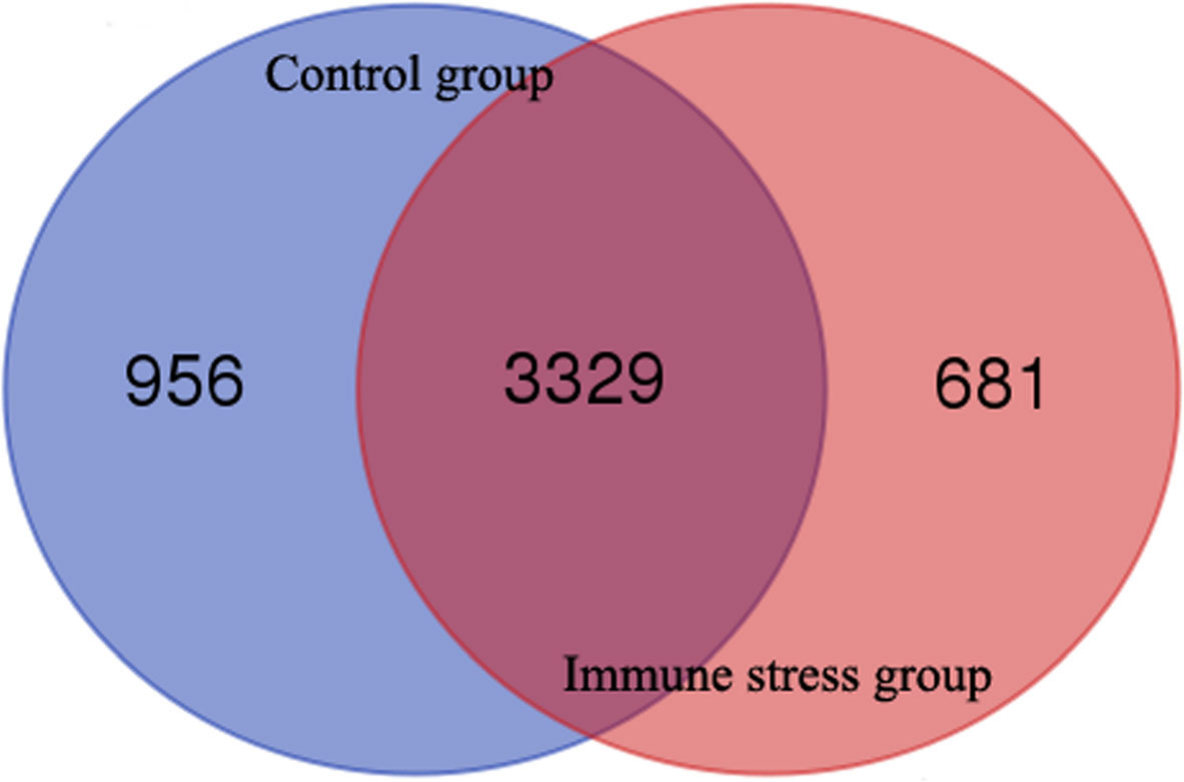
Venn diagram of the number of proteins expressed in the liver of broiler chickens in the control group and immune stress group

In the present study, a total of 4,966 proteins were identified in the liver tissues of broiler chickens. In the control group, 4,285 proteins (2,307 groups) were identified and 4,010 proteins (2,182 groups) were identified in the LPS group. As shown in Figs. 1, 3,329 proteins were expressed in both the control and treatment groups.

#### GO and KEGG analysis of unique proteins specially expressed in the control group

**Fig. 2:**
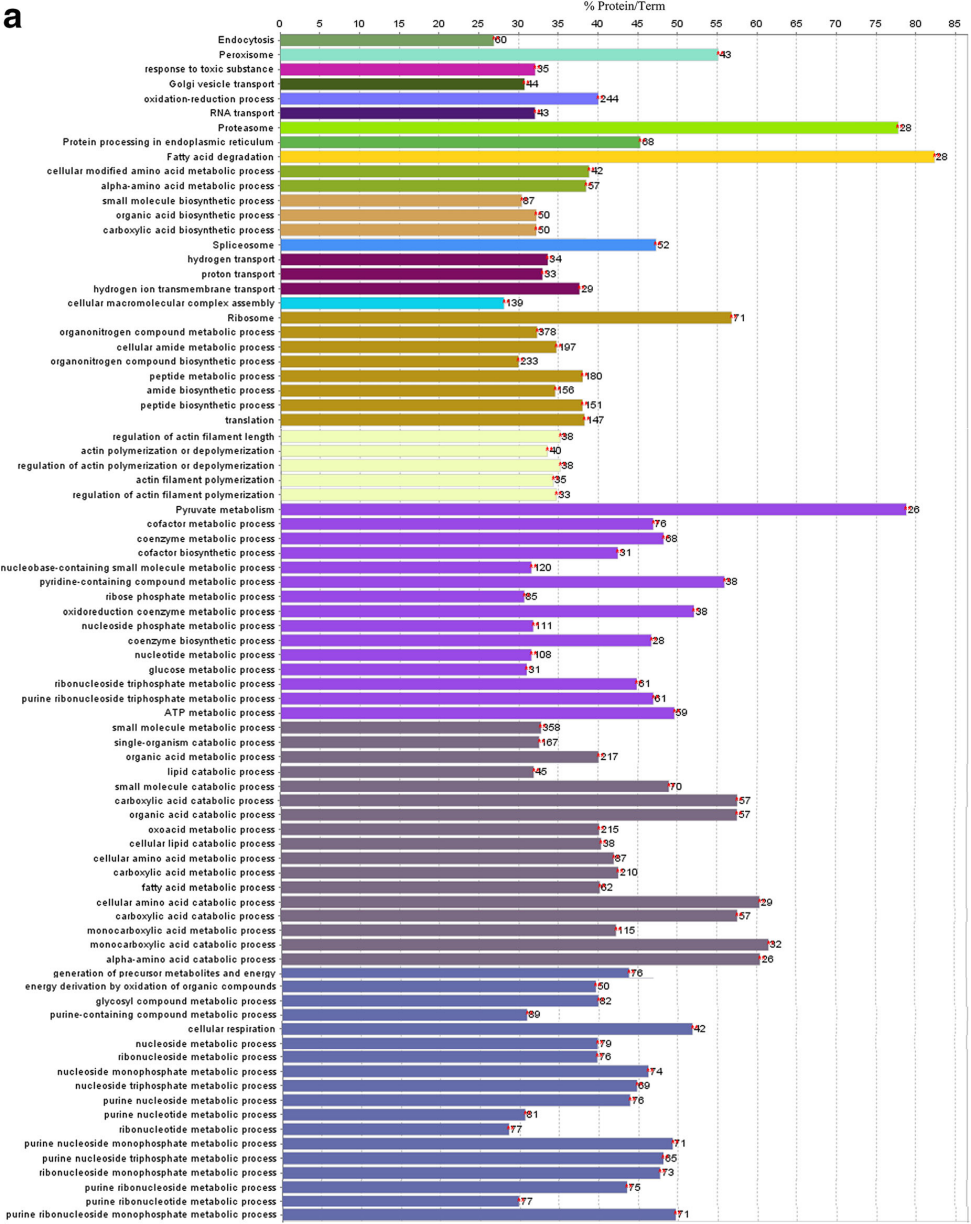

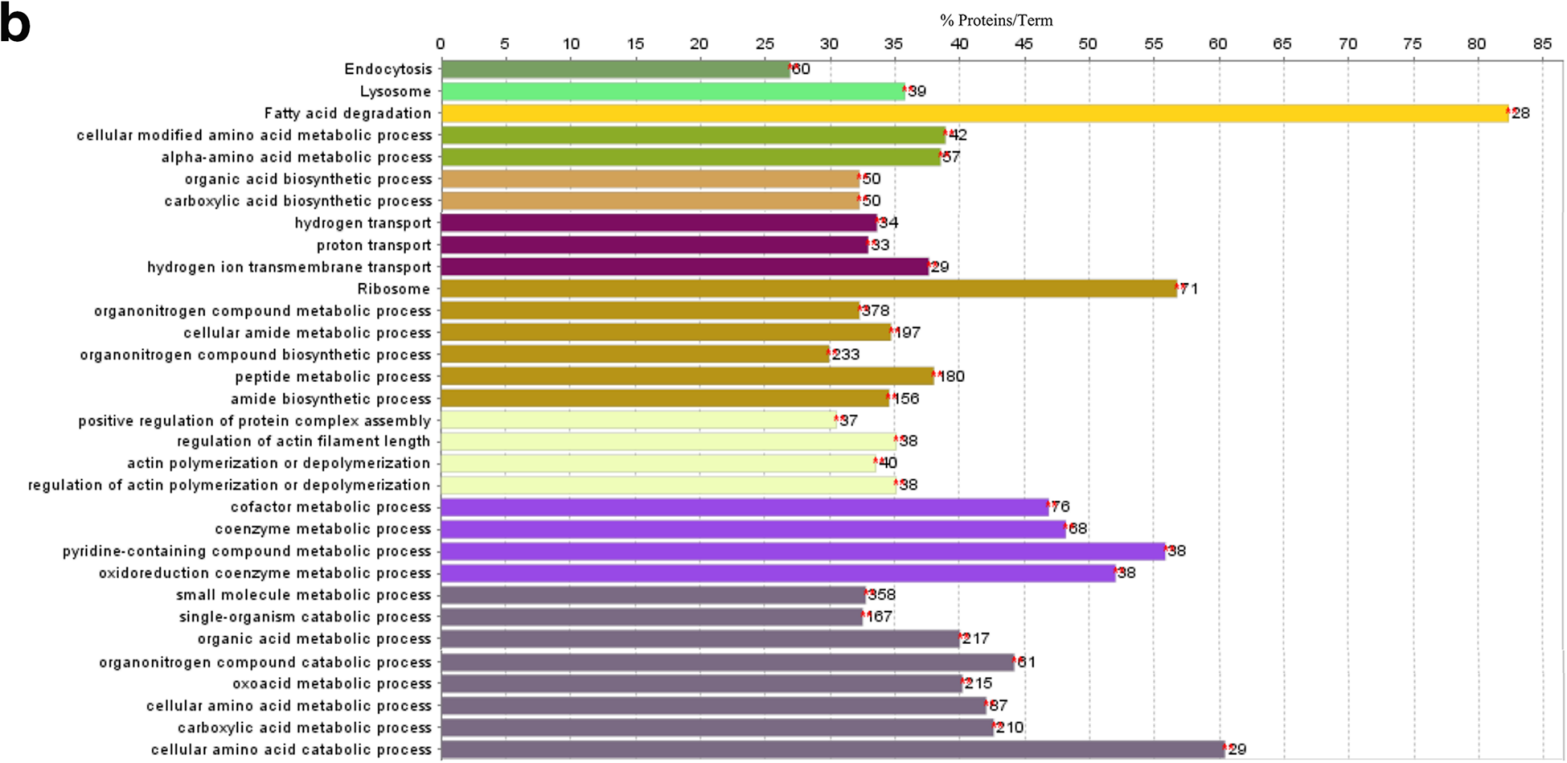
Qualitative proteome comparisons of the liver of broiler chickens in the control group and the immune stress group. **a** and **b**, GO and KEGG annotation of unique proteins specifically expressed in the control group and the immune stress group, respectively. Terms that begin with an uppercase or lowercase letters are KEGG or GO annotation, respectively. % Proteins/Term stands for the proportion of genes enriched in corresponding functional groups. The bars with the same color represent the same functional groups they belong to. The numbers stand for the genes enriched to the corresponding functional groups

As shown in Fig. 2a, KEGG pathway analysis was performed on specifically expressed proteins in the control group and demonstrated enrichment of endocytosis, peroxisome, Golgi vesicle transport, RNA transport, proteasome, protein processing in endoplasmic reticulum, fatty acid degradation, spliceosome, ribosome and pyruvate metabolism pathways.

Go analysis showed that the following biological processes were enriched in the control group, including, response to toxic substances, oxidation-reduction, amino acid metabolism, small molecule biosynthesis, transportation (hydrogen or proton transport), proteins biosynthesis (organonitrogen compound metabolic and biosynthetic processes, translation), actin polymerization or depolymerization and its regulation, nucleic acid biosynthesis and metabolism (nucleoside phosphate metabolic process, nucleoside biosynthetic process, nucleoside monophosphate metabolic process etc.), fatty acid metabolism (fatty acid metabolic process and lipid catabolic process, etc.), cofactor and coenzyme biosynthetic and metabolic process, organic acid metabolism (organic acid catabolic or biosynthetic process, carboxylic acid, monocarboxylic acid and glycosyl compound metabolic or biosynthetic process).

#### GO and KEGG analysis of unique proteins specially expressed in the immune stress group

As shown in Fig. 2b, KEGG pathway analysis was performed on specifically expressed proteins in the immune stress group. Endocytosis, lysosome, fatty acid degradation, ribosome pathways were enriched.

Go analysis showed that the following biological processes were enriched in the LPS group, including, amino acid metabolism, organic acid and carboxylic acid biosynthesis, transportation (hydrogen or proton transport), organonitrogen compound metabolic and biosynthetic processes, positive regulation of protein complex assembly, actin polymerization or depolymerization and its regulation, cofactor and coenzyme metabolism, organic acid metabolism (organic acid, carboxylic acid, oxoacid and amino acid metabolic and catabolic processes).

### Quantitative differential analysis of hepatic proteome in broiler chickens between the control and the immune stress group

Label free LC-MS/MS quantitative analysis isolated 111 proteins that were differently expressed in the liver of broilers in the control and immune stress groups. Of the proteins, 83 were up-regulated, but 28 proteins were down-regulated in the immune stress group (Table 4). Down-regulated proteins in the immune stress group were not significantly enriched in GO terms.

**Table 4:** Protein information of differential abundance identified in the liver of AA broilers challenged with LPS

Protein	Accession no.	Symbol ID	Sequence coverage, %	#Unique peptide	Fold change
3-hydroxyisobutyryl-CoA hydrolase mitochondrial isoform X1	gi|971404063	HIBCH	13	1	0.080
Transferase CAF17 mitochondrial	gi|303227895	IBA57	4	1	0.082
Protein-glutamine gamma-glutamyltransferase 4	gi|57530757	TGM4	5	3	0.084
Serine protease inhibitor Kazal-type 2 isoform X2	gi|971393739	SPINK2	14	1	0.130
Eukaryotic translation elongation factor 1 epsilon-1	gi|971382396	EEF1E1	7	1	0.147
Cathelicidin-3 precursor	gi|906847364	CATHL3	19	2	0.156
L-amino-acid oxidase precursor	gi|372266150	IL4I1	5	2	0.157
Chromodomain-helicase-DNA-binding protein 5 isoform X5	gi|971429121	CHD5	1	1	0.171
BTB/POZ domain-containing protein KCTD12	gi|971377767	KCTD12	4	1	0.193
Cathelicidin-2 precursor	gi|403224971	CATHL2	40	4	0.194
TBC1 domain family member 10A	gi|971422101	TBC1D10A	3	1	0.196
2-amino-3-carboxymuconate-6-semialdehyde decarboxylase isoform X2	gi|971406099	ACMSD	6	1	0.202
Ribonuclease homolog precursor	gi|56118294	RSFR	11	1	0.202
T-cell immunoglobulin and mucin domain-containing protein 4 precursor	gi|57524995	TIMD4	6	2	0.212
Gallinacin-2 isoform X1	gi|971390683	GAL2	12	1	0.219
Lymphocyte antigen 86 precursor	gi|52138689	LY86	9	1	0.220
28S ribosomal protein S22 mitochondrial	gi|971410189	MRPS22	3	1	0.261
Ubiquilin-1 isoform X1	gi|118104137	UBQLN1	1	1	0.270
Serine/threonine-protein kinase 4 isoform X1	gi|971427386	STK4	2	1	0.272
Myeloid protein 1 precursor	gi|758818508	MIM1	32	8	0.273
FAS-associated death domain protein	gi|118091445	FADD	7	1	0.280
Protein MRP-126	gi|760997140	S100A9	25	3	0.304
Lysozyme g precursor	gi|47825389	LYG2	11	2	0.313
PREDICTED: Acetyl-CoA carboxylase isoform X2	gi|971425697	ACAC	4	6	0.322
Phosphomannomutase 2	gi|71895479	PMM2	4	1	0.324
Hydroxyacid-oxoacid transhydrogenase mitochondrial isoform X1	gi|971384192	ADHFE1	8	2	0.333
Serine/arginine-rich splicing factor 2	gi|47604918	SRSF2	8	1	0.337
Tyrosine-protein kinase Lyn	gi|57530388	LYN	3	1	0.347
Trifunctional purine biosynthetic protein adenosine-3	gi|47825387	GART	5	1	0.351
59 kDa 2′-5′-oligoadenylate synthase-like protein isoform X1	gi|971415867	OASL	3	1	0.351
Antigen peptide transporter 2 isoform X1	gi|971422259	TAP2	2	1	0.359
Dynein light chain roadblock-type 1 isoform X1	gi|971427252	DYNLRB1	12	1	0.372
Gallinacin-7 preproprotein	gi|48976031	AvBD7	15	1	0.378
Fibrinogen gamma chain precursor	gi|766944255	FGG	41	13	0.386
Splicing factor 3B subunit 6	gi|50745107	SF3B6	10	1	0.387
Pre-mRNA-processing factor 39 isoform X1	gi|363734910	PRPF39	2	1	0.387
Glutaredoxin-1	gi|45384038	GLRX	10	1	0.387
Phosphoglucomutase-2	gi|71897287	PGM2	4	1	0.389
Chloride intracellular channel protein 2	gi|71895359	CLIC2	16	2	0.390
Metallothionein-3	gi|147901436	MT3	19	1	0.393
Fructose-bisphosphate aldolase C	gi|330417943	ALDOC	23	4	0.401
GTP cyclohydrolase 1 feedback regulatory protein	gi|313747529	GCHFR	20	1	0.405
Proteasome subunit alpha type-3	gi|57529899	PSMA3	5	1	0.415
Heterogeneous nuclear ribonucleoprotein M isoform X2	gi|971435624	HNRNPM	8	1	0.416
Protein phosphatase 1 regulatory subunit 42 isoform X2	gi|118087042	PPP1R42	3	1	0.422
Sorcin	gi|124249424	SRI	12	2	0.425
Gallinacin-1 alpha precursor	gi|45384510	GAL1	12	1	0.428
Fibrinogen beta chain precursor	gi|267844833	FGB	37	13	0.437
Acid ceramidase precursor	gi|57530079	ASAH1	3	1	0.438
Cytochrome P450 2D3-like	gi|307078128	CYP2D6	2	1	0.447
Antigen peptide transporter 1	gi|209863064	TAP1	2	1	0.450
Integrin alpha-V-like	gi|971443478	LOC107056639	7	1	0.458
Protein O-GlcNAcase isoform X1	gi|971402723	MGEA5	1	1	0.458
Dihydropteridine reductase	gi|57529509	QDPR	16	2	0.461
RNA-binding protein 39 isoform X2	gi|513217267	RBM39	2	1	0.461
Neutral alpha-glucosidase AB-like	gi|971451761	LOC107051327	9	1	0.464
Complement 4 precursor	gi|116175422	C4	2	2	0.466
Fibrinogen alpha chain isoform 1 precursor	gi|429484490	FGA	13	6	0.468
Guanylate-binding protein 1-like	gi|971415657	GBP4L	2	1	0.470
F-box only protein 6	gi|971429573	FBXO6	4	1	0.471
Erythroblast NAD(P)(+)--arginine ADP-ribosyltransferase isoform X2	gi|513166631	ART7C	3	1	0.473
Carbonyl reductase [NADPH] 1 isoform X1	gi|971375185	CBR1	30	5	0.482
D-2-hydroxyglutarate dehydrogenase mitochondrial	gi|971410344	D2HGDH	2	1	0.485
Protein LOC107050412	gi|971449891	LOC107050412	4	1	0.487
Protein GIMAP1	gi|971379314	GIMAP1	1	1	0.487
Peroxisomal trans-2-enoyl-CoA reductase	gi|57529732	PECR	7	1	0.495
Filamin-B isoform X1	gi|971415729	FLNB	2	2	0.505
D-dopachrome decarboxylase	gi|71897241	DDT	11	1	0.508
V-type proton ATPase subunit E 1	gi|57525423	ATP6V1E1	12	2	0.511
Choline dehydrogenase mitochondrial isoform X1	gi|513205065	CHDH	1	1	0.526
ATP-dependent RNA helicase DDX24	gi|971400168	DDX24	1	1	0.527
26S proteasome non-ATPase regulatory subunit 9	gi|57525182	PSMD9	6	1	0.534
NADH dehydrogenase [ubiquinone] iron-sulfur protein 3 mitochondrial isoform X2	gi|971398316	NDUFS3	9	2	0.540
Alanine--glyoxylate aminotransferase 2 mitochondrial	gi|513228840	AGXT2	7	2	0.553
Ornithine aminotransferase mitochondrial	gi|57529515	OAT	4	1	0.561
Metallothionein	gi|46048711	MT1	43	3	0.576
60S ribosomal protein L19	gi|71896335	RPL19	9	1	0.589
Epididymal secretory protein E1 precursor	gi|71894903	NPC2	14	1	0.603
Long-chain fatty acid transport protein 4	gi|971423093	SLC27A4	2	1	0.616
Glutaredoxin-3	gi|475506756	GLRX3	4	1	0.617
Cytoplasmic FMR1-interacting protein 1 isoform X2	gi|971376600	CYFIP1	1	1	0.624
Diamine acetyltransferase 2-like	gi|971451573	LOC107051219	23	2	0.631
Platelet glycoprotein 4	gi|71897003	CD36	3	1	0.646
Ras-related protein Rab-10	gi|71895051	RAB10	6	1	1.543
Iron-sulfur cluster assembly enzyme ISCU mitochondrial	gi|971421427	ISCU	11	1	1.574
Nucleophosmin	gi|45383996	NPM1	22	5	1.601
40S ribosomal protein S11	gi|71895103	RPS11	15	2	1.654
UBX domain-containing protein 4 isoform X4	gi|513195319	UBXN4	3	1	1.757
C-factor-like isoform X2	gi|363738106	LOC415662	38	6	1.765
Sodium-coupled neutral amino acid transporter 4 isoform X1	gi|971370947	SLC38A4	2	1	2.005
UDP-glucose 4-epimerase	gi|363742411	GALE	3	1	2.086
Protein disulfide-isomerase A4	gi|57530768	PDIA4	7	5	2.091
Band 4.1-like protein 2 isoform X4	gi|971387884	EPB41L2	1	1	2.092
NADH dehydrogenase [ubiquinone] 1 beta subcomplex subunit 1	gi|480540334	NDUFB1	19	1	2.118
Granulysin precursor	gi|113206146	GNLY	30	3	2.139
Aspartyl aminopeptidase	gi|61098378	DNPEP	4	2	2.149
GTPase IMAP family member 5	gi|971379352	GIMAP5	4	1	2.180
Regucalcin isoform X1	gi|971376431	RGN	30	7	2.180
ATP-citrate synthase isoform X3	gi|971435352	ACLY	6	5	2.206
Glutathione S-transferase alpha 4 isoform X1	gi|971389828	GSTA4L	10	2	2.241
Adrenodoxin mitochondrial	gi|310832417	FDX1	5	1	2.574
Protein syndesmos precursor	gi|45382147	SDC4	8	2	2.723
Interferon alpha-inducible protein 6	gi|47777293	IFI6	89	6	3.222
Regulator of microtubule dynamics protein 1	gi|475808820	RMDN1	22	6	3.671
Proteasome subunit beta type-7	gi|45383366	PSMB7	5	1	3.850
Tetratricopeptide repeat protein 38-like	gi|363728070	TTC38L	3	1	4.244
Glutathione S-transferase theta-1-like	gi|971421234	GSTT1L	11	2	4.318
ADP/ATP translocase 1	gi|57530120	SLC25A4	7	2	5.735
Interferon-induced GTP-binding protein Mx	gi|45382939	MX	5	2	5.960
Cytochrome P450 2H2 precursor	gi|48976111	CYP2C23b	28	11	6.432
Trifunctional purine biosynthetic protein adenosine-3 isoform X1	gi|971375154	GART	5	4	+∞

**Fig. 3:**
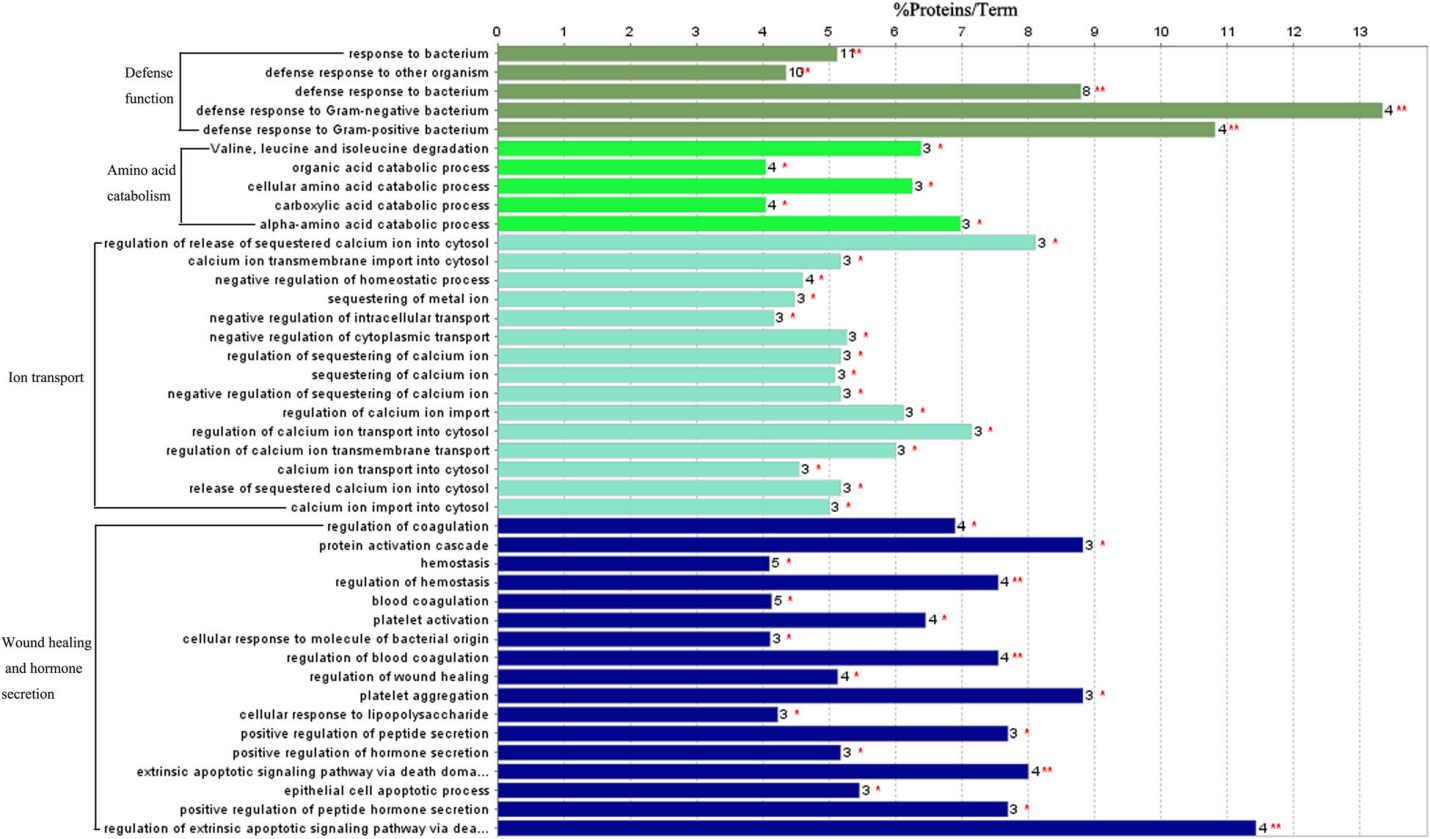
GO and KEGG annotation of up-regulated proteins in the liver of broilers chickens in the immune stress group. Terms that begin with an uppercase or lowercase letters are KEGG or GO annotation. % Proteins/Term stands for the proportion of genes enriched in corresponding functional groups. The bars with the same color represent the same functional groups they belong to. The numbers stand for the genes enriched to the corresponding functional groups

As Fig. 3 and Table 5 show, up-regulated proteins in the immune stress group were significantly enriched in GO terms of defense function, amino acid catabolism, ion transport and regulation, wound healing and hormone secretion and regulation. More specifically, up-regulated proteins in the immune stress group were enriched in valine, leucine and isoleucine degradation pathways. However, there were no GO terms and pathways enriched in down-regulated proteins of the immune stress group.

**Table 5:** GO annotation of upregulated proteins in broiler chickens in the immune stress group

GO ID	GO Term	Term *P*- value	Associated proteins enriched
Biological process			
GO:0009617	Response to bacterium	81.0E-9	AvBD1, AvBD2, AvBD7, CATH2, CATH3, CD36, FGB, LY86, LYN, RSFR, TAP2
GO:0098542	Defense response to other organism	1.4E-6	AvBD1, AvBD2, AvBD7, CATH2, CATH3, CD36, FADD, FGB, OASL, RSFR
GO:0042742	Defense response to bacterium	87.0E-9	AvBD1, AvBD2, AvBD7, CATH2, CATH3, CD36, FGB, RSFR
GO:0050829	Defense response to Gram-negative bacterium	33.0E-6	AvBD7, CATH2, CATH3, RSFR
GO:0050829	Defense response to Gram-positive bacterium	79.0E-6	CATH2, CATH3, CD36, RSFR
GO:0016054	Organic acid catabolic process	3.3E-3	AGXT2, HIBCH, OAT, SLC27A4
GO:0009063	Cellular amino acid catabolic process	3.3E-3	AGXT2, HIBCH, OAT
GO:0046395	Carboxylic acid catabolic process	3.3E-3	AGXT2, HIBCH, OAT, SLC27A4
GO:1901606	Alpha-amino acid catabolic process	2.4E-3	AGXT2, HIBCH, OAT
GO:0051279	Regulation of release of sequestered calcium ion into cytosol	1.5E-3	CLIC2, LYN, SRI
GO:0097553	Calcium ion transmembrane import into cytosol	5.6E-3	CLIC2, LYN, SRI
GO:0032845	Negative regulation of homeostatic process	2.1E-3	CLIC2, FADD, LYN, SRI
GO:0051238	Sequestering of metal ion	8.4E-3	CLIC2, LYN, SRI
GO:0032387	Negative regulation of intracellular transport	10.0E-3	CD36, CLIC2, SRI
GO:1903650	Negative regulation of cytoplasmic transport	5.3E-3	CD36, CLIC2, SRI
GO:0051282	Regulation of sequestering of calcium ion	5.6E-3	CLIC2, LYN, SRI
GO:0051208	Sequestering of calcium ion	5.9E-3	CLIC2, LYN, SRI
GO:0051283	Negative regulation of sequestering of calcium ion	5.6E-3	CLIC2, LYN, SRI
GO:0090279	Regulation of calcium ion import	3.5E-3	CLIC2, LYN, SRI
GO:0010522	Regulation of calcium ion transport into cytosol	2.2E-3	CLIC2, LYN, SRI
GO:1903169	Regulation of calcium ion transmembrane transport	3.7E-3	CLIC2, LYN, SRI
GO:0060402	Calcium ion transport into cytosol	8.0E-3	CLIC2, LYN, SRI
GO:0051209	Release of sequestered calcium ion into cytosol	5.6E-3	CLIC2, LYN, SRI
GO:1902656	Calcium ion import into cytosol	6.2E-3	CLIC2, LYN, SRI
GO:0050818	Regulation of coagulation	460.0E-6	CD36, FGB, FGG, LYN
GO:0072376	Protein activation cascade	1.2E-3	C4, FGB, FGG
GO:0007599	Hemostasis	970.0E-6	CD36, FGA, FGB, FGG, LYN
GO:1900046	Regulation of hemostasis	320.0E-6	CD36, FGB, FGG, LYN
GO:0007596	Blood coagulation	930.0E-6	CD36, FGA, FGB, FGG, LYN
GO:0030168	Platelet activation	590.0E-6	FGA, FGB, FGG, LYN
GO:0071219	Cellular response to molecule of bacterial origin	10.0E-3	CD36, LY86, LYN
GO:0030193	Regulation of blood coagulation	320.0E-6	CD36, FGB, FGG, LYN
GO:0061041	Regulation of wound healing	1.4E-3	CD36, FGB, FGG, LYN
GO:0070527	Platelet aggregation	1.2E-3	FGB, FGG, LYN
GO:0071222	Cellular response to LPS	9.8E-3	CD36, LY86, LYN
GO:0002793	Positive regulation of peptide secretion	1.8E-3	FGB, FGG, SRI
GO:0046887	Positive regulation of hormone secretion	5.6E-3	FGB, FGG, SRI
GO:0008625	Extrinsic apoptotic signaling pathway via death domain receptors	250.0E-6	FADD, FGB, FGG, STK4
GO:1904019	Epithelial cell apoptotic process	4.8E-3	FGB, FGG, STK4
GO:0090277	Positive regulation of peptide hormone secretion	1.8E-3	FGB, FGG, SRI
GO:1902041	Regulation of extrinsic apoptotic signaling pathway via death domain receptors	63.0E-6	FADD, FGB, FGG, STK4
Pathway			
GO:0000280	Valine, leucine and isoleucine degradation	3.1E-3	AGXT2, HIBCH, IL4I1

As shown in Table 6, LPS binding was enriched in up-regulated proteins in the immune stress group using GO annotation based on the molecular function cluster. Moreover, up-regulated proteins in the immune stress group were distributed in the extracellular region, fibrinogen complex, secretory granule, extracellular space and cytoplasm, respectively.

**Table 6:** GO annotation of upregulated proteins in broilers chickens in the immune stress group based on molecular function and cellular component

Molecular function			
GO Term	Description	Count in gene set	False discovery rate
GO:0001530	LPS binding	2 of 4	0.0389
Cellular component			
GO:0005576	Extracellular region	12 of 299	1.08e-06
GO:0005577	Fibrinogen complex	2 of 2	0.0021
GO:0030141	Secretory granule	3 of 23	0.0027
GO:0005615	Extracellular space	5 of 167	0.0093
GO:0005737	Cytoplasm	13 of 1125	0.0149

### Protein and protein interaction (PPI) analysis of differentially expressed proteins in the immune stress group

**Fig. 4:**
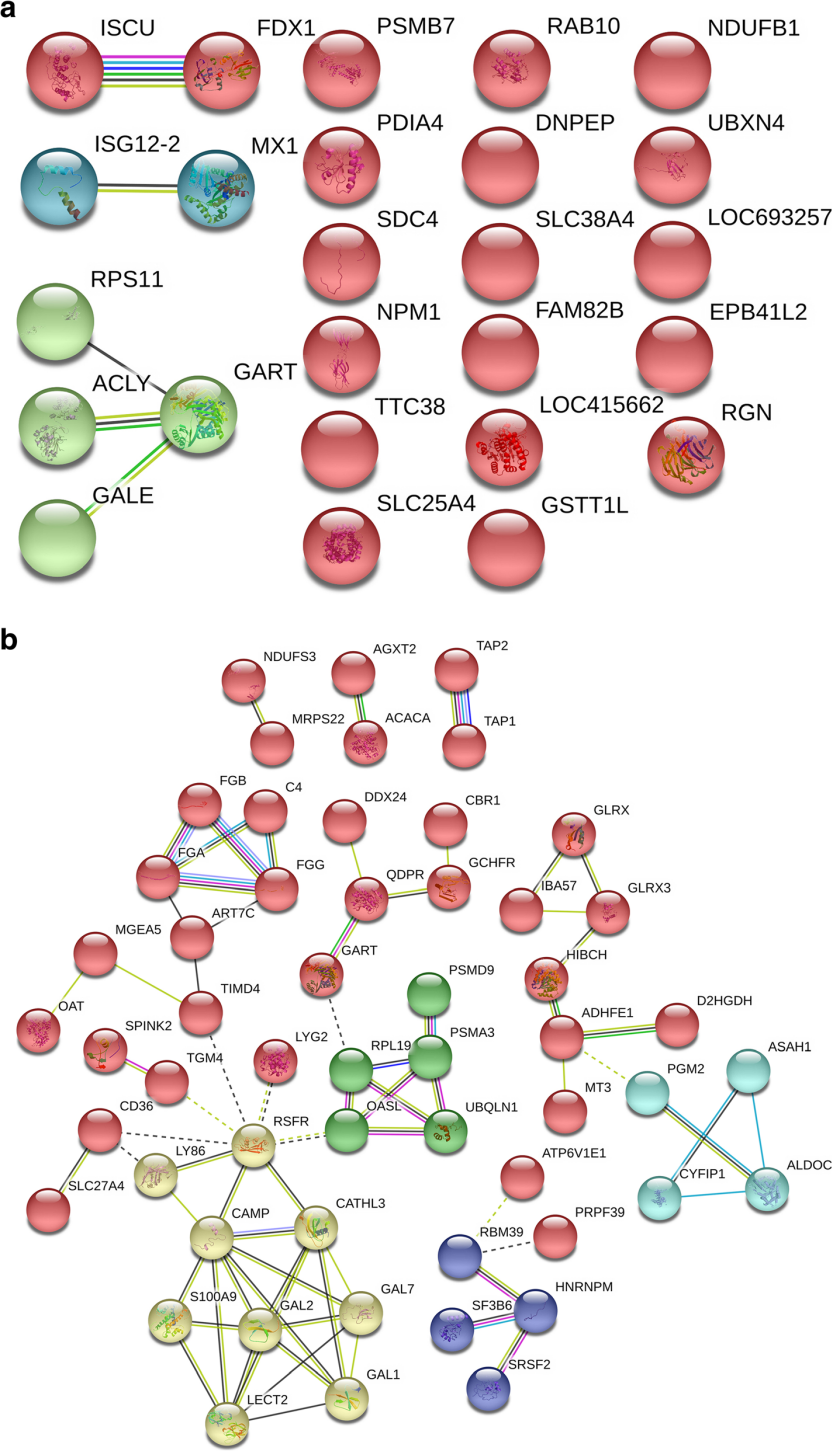
Protein and protein interaction network of differentially expressed proteins in the liver of broilers chickens in the immune stress group. A and B represent interaction network of down-regulated and up-regulated proteins in the liver of broilers chickens in the immune stress group, respectively .Each ball represents node protein, the same color balls represent node proteins clustered in the same sub network. The solid line indicates that the interaction score between the two proteins is more than 0.5 (the dotted line indicates that the score is less than 0.5). Different color solid lines between proteins represent evidence of association. Red lines indicate fusion evidence, green lines indicate neighborhood evidence, blue lines indicate co-occurrence evidence, purple lines indicate experimental evidence, yellow lines indicate text mining evidence, light blue lines indicate database evidence, and black lines indicate co-expression evidence

PPI analysis showed that there are only eight proteins connected to the network, including iron-sulfur cluster assembly enzyme (ISCU), adrenodoxin (FDX1), interferon alpha-inducible protein 6 (ISG12–2), interferon-induced GTP-binding protein Mx (MX1), 40S ribosomal protein S11 (RPS11), ATP-citrate synthase isoform X3 (ACLY), UDP-glucose 4-epimerase (GALE), trifunctional purine biosynthetic protein adenosine-3 isoform X1 (GALE). However, there is no significant interaction network (*P* = 0.248), as Fig. 4a shows.

The results of PPI analysis of upregulated proteins in the immune stress group showed that 77 proteins were connected into the networks with significant interaction between the networks (*P* = 6.84E-11), as shown in Fig. 4b. Furthermore, cluster analysis showed that the whole network was interconnected by 5 sub-networks, involved in defense function (yellow nodes), protein biosynthesis (Green nodes), RNA splicing and binding (Blue nodes), carboxylic acid metabolism (Cyan) and nutrient metabolism (Red nodes).

## Discussion

Immune stress resulting from a LPS challenge inhibited the growth of broilers in this study. The study shows that the concentrations of IL-1β, TNF-α and IL-6 in the serum of broilers injected with LPS was significantly increased. These inflammatory cytokines triggered an up-regulation of the expression of hepatic proteins involved in the immune defense function, amino acid catabolism, ion transport and wound healing and hormone secretion. Moreover, the data revealed that immune stress enhanced the secretion of ACTH and CORT but decreased the secretion of GH and IGF-1. Furthermore, immune stress enhanced hepatic degradation pathways for valine, leucine and isoleucine which would contribute to the growth depression noted by many authors following an immunological challenge [4, 10, 11, 13–15].

### Immune stress enhanced the expression of proteins related to defense function

Inflammatory cytokines such as IL-1 and IL-6 can activate B cells and trigger the humoral immune response. Studies have shown that increased humoral response is associated with the inflammatory response [13, 28]. In our study, the serum concentrations of IL-1β and IL-6, following LPS injection, were significantly higher than in unchallenged broilers. Moreover, the present experiment showed that immune stress enhanced the expression of defense function proteins (GO:0009617, GO:0098542, GO:0042742, GO:0050829, GO:0050829), including AvBD1, AvBD2, AvBD7, CATH2, CATH3, CD36, FGB, LY86, LYN, RSFR, TAP2, FADD, and OASL.

These upregulated proteins include effector proteins expressed to directly inactivate pathogens or proteins protecting the chicken’s own tissues against damage. Heterophils are responsible for pathogen inactivation by the release of two classes of antimicrobial peptides, i.e. cathelicidins CATHL1, CATHL2, CATHL3 and gallinacins GAL1, GAL2 and GAL7 (also called avian β-defensins AvBD1, AvBD2 and AvBD7) [29]. These proteins are present in the granules of chicken heterophils associated with response to Salmonella infection [30, 31]. RSFR exhibits multiple enzymatic activities and as a ribonuclease A, it has angiogenic and bactericidal properties [32]. The angiogenic potential of RSFR facilitates the restoration of damaged tissues following inflammation. The bactericidal effects of RFSR protein and its modulatory effect on dendritic cells polarises the immune response towards a Th2 response in chickens [33]. Therefore hepatic upregulation of RSFR, as observed in the immune stress group, suggests that RSFR could contribute to both tissue repair and clearance of residual bacterial pathogens.

### Immune stress up-regulated the expression of proteins related to wound healing

Immune stress can lead to delayed wound healing [9]. Up-regulated proteins include those involved in LPS neutralisation and healing of host tissue. In this study, LPS binding (GO:0001530, GO:0071219, GO:0071222) was enriched in GO analysis based on molecular function, including CATHL2, LY86 and complement proteins. Tyrosine-protein kinase Lyn (LYN) plays a role in the LPS-mediated signaling pathway, and in positive regulation of the stress-activated protein kinase signaling cascade. CD36 is involved in the cell surface receptor signaling pathway. Complement 4 precursor is also a defense protein (C4) [34, 35]. Chicken heterophils express lysozyme and two classes of antimicrobial peptides, i.e. cathelicidins and gallinacins. Besides pathogen inactivation, chicken heterophils are also involved in tissue protection and wound healing (GO:0061041) by the expression of RSFR, TGM4, CD36, FGB, FGG and LYN.

Transglutaminases TGM_3_ and TGM_4_, are also induced during inflammation [36]. Interestingly, transglutaminase inhibitor cystamine reduced the inflammation induced by 2,4,6-trinitrobenzene sulfonic acid in rats [37]. Transglutaminases catalyse the formation of an isopetide bond between the carboxyamide group of glutamine and the ε amino group of lysine leading to protein cross-linking. TGM_3_ was induced in the lungs of pigs experimentally infected with *Salmonella choleraesuis* [38]. TGM_3_ can cross-link with other proteins during wound healing. In chickens, transglutaminase TGM_4_ is expressed in B-lymphocytes and to a lesser extent in macrophages [35] and may have a function in wound healing. This would explain up-regulation of TGM_4_ in the liver of broiler chickens challenged by LPS.

As a consequence of the immune response, blood coagulation is often exploited by pathogens for reason of infective and septic processes. For coagulation, this trigger is usually some form of vascular injury, followed by activation. In the classical waterfall model, each activated protein goes on to activate the next protein in a rapidly expanding cascade of reactions which quickly results in the local formation of a fibrin clot to seal the injury [39]. For example, FG are targeted by bacteria, thus offering a straightforward explanation of positive selection. FG is comprised of the α, β, and γ genes of fibrinogen (FG) (FGA, FGB, and FGG) [40]. In mammals, fibrin (ogen) also serves as a platform for migrating cells, can act as a chemoattractant, and regulates inflammation by activating immune cells, especially macrophages [41]. In the avian thymus, genes encoding fibrinogen subunits (FGA, FGG and FGB) were among the most significantly expressed genes in the broiler after exposure to heat stress and LPS treatments [42]. The present study showed that the biological processes (GO:0050818, GO:0072376, GO:0007599, GO:1900046, GO:0007596, GO:0030168, GO:0030193 and GO:0070527) were enriched, including FGA, FGG and FGB which were up-regulated in the liver of broilers challenged with LPS. This suggests that chickens stimulated by LPS were constantly triggering their body systems to “heal the damage”.

### Immune stress enhanced the expression of proteins related to amino acid catabolism

KEGG pathway analysis indicated that the valine, leucine and isoleucine degradation pathway (GO:0000280) was significantly enriched, involving AGXT2, HIBCH, IL4I1. IL4I1 that also play a role in the L-phenylalanine catabolic process. IL4I1 was up-regulated in the spleen [22], the bursa of Fabricius [43] and the thymus gland when birds were exposed to LPS [44]. In this study, IL4I1 was up-regulated in the liver of broilers challenged with LPS. HIBCH is involved in L-valine degradation. AGXT2 plays a role in the glyoxylate catabolic process, L-alanine catabolic process, glycine biosynthetic process and regulation of nitric oxide biosynthesis. Enhancing organic acid catabolism processes (GO:0016054, GO:0009063, GO:0046395, GO:1901606) confirms that body protein and fat anabolism will be reduced by immune stress, resulting in lower feed utilisation and decreased growth performance [14].

OAT has ornithine-oxo-acid transaminase activity and is associated with L-proline biosynthesis. SLC27A4 positively regulates serine/threonine kinase activity and participate in phosphatidylcholine biosynthesis. Up-regulated expression of OAT and SLC27A4 indicates that catabolism will be enhanced in order to meet the nutrients required to synthesize immune effector molecules. This repartitioning of nutrients away from growth and development will reduce bird productivity [15].

### Immune stress upregulated the expression of ion transport proteins

Cells of the innate and adaptive immune systems express various ion transporters that allow the influx and efflux of ions across the plasma membrane or their release from intracellular organelles such as the endoplasmic reticulum (ER), mitochondria, and lysosomes [45]. Stimulation of antigen receptors results in a rapid increase in Ca^2+^ originating from the ER and the extracellular space through PM Ca^2+^ channels that is required for sustained Ca^2+^ elevations [46]. SRI is involved in the regulation of high voltage-gated calcium channel activity. CLIC2 is related to the regulation and release of sequestered Ca^2+^ into the cytosol by sarcoplasmic reticulum. Ca^2+^ signals also mediate T cell motility. In this study, the up-regulation of proteins associated with ion transport (GO:0051279, GO:0097553, GO:0032845, GO:0051238, GO:0032387, GO:1903650, GO:0051282, GO:0051208, GO:0051283, GO:0090279, GO:0010522, GO:1903169, GO:0060402, GO:0051209 and GO:1902656) suggests that immune stress could trigger the innate and adaptive immune function by inducing the hepatic expression of SRI, CLIC2 and LYN in broilers.

### Immune stress increased the expression of proteins related to hormone secretion

When under immune stress, excessive inflammatory cytokines may lead to the activation of the HPA axis, increasing the secretion of ACTH and CORT, and reducing the secretion of the growth promoting hormones such as GH and IGF-1 [47]. In our experiment, function enrichment analysis of up-regulated proteins showed the positive regulation of peptide and hormone secretion (GO:0090277 and GO:0046887) and positive regulation of peptide secretion (GO:0002793) were enriched, including FGB, FGG and SRI.

In the lymphocyte life cycle, T and B cells numbers will be reduced through apoptosis at different stages of ontological development of the immune system to avoid the accumulation and the potential for autoimmunity. However, apoptosis induced by external factors, such as vaccination-induced stress, would cause adverse responses that affect growth performance. It has been shown that stress can trigger the apoptosis of pre-B cells by inducing high concentrations of glucocorticoid, resulting in the reduction of the number of B lymphocytes and suppressed immunity. It has been determined that the infectious bursal disease vaccine can induce apoptotic effects in the bursa of Fabricius [48]. Studies have shown that serum ACTH and CORT concentrations significantly increase due to immune stress induced by LPS [7, 49], and the elevated concentrations of serum ACTH and CORT in these studies are consistent with those observed in the current experiment. High concentrations of ACTH and CORT induces apoptotic effects in spleen lymphocytes [49]. Consistent with these studies, our results show that when apoptosis is induced, enhanced expression of proteins related to the apoptotic signaling pathway (GO:0008625 and GO:1902041) and cell apoptotic process (GO:1904019), involving FADD, FGB, FGG, STK4.

## Conclusions

The immune stress induced by LPS triggered alterations in the hepatic proteome of broiler chickens and provides a new insight into the mechanisms by which immune challenge impairs bird growth or productivity. In this regard, impaired growth is secondary to reduced feed intake, which has been well described in the literature [4, 15], and the repartitioning of nutrients. We have demonstrated at a molecular level, that immune stress redirects nutrients which were destined for muscle synthesis and growth to the immune system to support increased functionality. This was evident from increased expression of hepatic proteins involved in defense function, amino acid catabolism, ion transport, wound healing, hormone secretion, and pathogen clearance. The activated immune system can resist immunological challenges but the additional nutritional and metabolic demands imposed on the bird can result in a decline in growth performance.

## Data Availability

The datasets used and analyzed during the current study are available from the corresponding author on reasonable request.

## Abbreviations

LC-MS: Label-free liquid chromatography and mass spectrometry
LPS: Lipopolysaccharide
AA: Arbor Acres
ACTH: Adrenocorticotropic hormone
CORT: Corticosterone
GH: Growth hormone
IGF-1: Insulin-like growth factor-1
IL-1β: Interleukin-1β
IL-6: Interleukin-6
TNF-α: Tumour necrosis factor-α
DTT: Dithiothreitol
TIC: Total ion current
GO: Gene Ontology
KEGG: Kyoto Encyclopedia of Genes and Genomes
HIBCH: 3-hydroxyisobutyryl-CoA hydrolase mitochondrial isoform X1
IBA57: Transferase CAF17 mitochondrial
TGM4: Protein-glutamine gamma-glutamyltransferase 4
SPINK2: Serine protease inhibitor Kazal-type 2 isoform X2
EEF1E1: Eukaryotic translation elongation factor 1 epsilon-1
CATHL3: Cathelicidin-3 precursor
IL4I1: L-amino-acid oxidase precursor
CHD5: Chromodomain-helicase-DNA-binding protein 5 isoform X5
KCTD12: BTB/POZ domain-containing protein KCTD12
CATHL2: Cathelicidin-2 precursor
TBC1D10A: TBC1 domain family member 10A
ACMSD: 2-amino-3-carboxymuconate-6-semialdehyde decarboxylase isoform X2
RSFR: Ribonuclease homolog precursor
TIMD4: T-cell immunoglobulin and mucin domain-containing protein 4 precursor
GAL2: Gallinacin-2 isoform X1
LY86: Lymphocyte antigen 86 precursor
MRPS22: 28S ribosomal protein S22 mitochondrial
UBQLN1: Ubiquilin-1 isoform X1
STK4: Serine/threonine-protein kinase 4 isoform X1
MIM1: Myeloid protein 1 precursor
FADD: FAS-associated death domain protein
S100A9: Protein MRP-126
LYG2: Lysozyme g precursor
ACAC: PREDICTED: acetyl-CoA carboxylase isoform X2
PMM2: Phosphomannomutase 2
ADHFE1: Hydroxyacid-oxoacid transhydrogenase mitochondrial isoform X1
SRSF2: Serine/arginine-rich splicing factor 2
LYN: Tyrosine-protein kinase Lyn
GART: Trifunctional purine biosynthetic protein adenosine-3
OASL: 59 kDa 2'-5'-oligoadenylate synthase-like protein isoform X1
TAP2: Antigen peptide transporter 2 isoform X1
DYNLRB1: Dynein light chain roadblock-type 1 isoform X1
AvBD7: Gallinacin-7 preproprotein
FGG: Fibrinogen gamma chain precursor
SF3B6: Splicing factor 3B subunit 6
PRPF39: Pre-mRNA-processing factor 39 isoform X1
GLRX: Glutaredoxin-1
PGM2: Phosphoglucomutase-2
CLIC2: Chloride intracellular channel protein 2
MT3: Metallothionein-3
ALDOC: Fructose-bisphosphate aldolase C
GCHFR: GTP cyclohydrolase 1 feedback regulatory protein
PSMA3: Proteasome subunit alpha type-3
HNRNPM: Heterogeneous nuclear ribonucleoprotein M isoform X2
PPP1R42: Protein phosphatase 1 regulatory subunit 42 isoform X2
SRI: Sorcin
GAL1: Gallinacin-1 alpha precursor
FGB: Fibrinogen beta chain precursor
ASAH1: Acid ceramidase precursor
CYP2D6: Cytochrome P450 2D3-like
TAP1: Antigen peptide transporter 1
LOC107056639: Integrin alpha-V-like
MGEA5: Protein O-GlcNAcase isoform X1
QDPR: Dihydropteridine reductase
RBM39: RNA-binding protein 39 isoform X2
LOC107051327: Neutral alpha-glucosidase AB-like
C4: Complement 4 precursor
FGA: Fibrinogen alpha chain isoform 1 precursor
GBP4L: Guanylate-binding protein 1-like
FBXO6: F-box only protein 6
ART7C: Erythroblast NAD(P)(+)--arginine ADP-ribosyltransferase isoform X2
CBR1: Carbonyl reductase [NADPH] 1 isoform X1
D2HGDH: D-2-hydroxyglutarate dehydrogenase mitochondrial
LOC107050412: Protein LOC107050412
GIMAP1: Protein GIMAP1
PECR: Peroxisomal trans-2-enoyl-CoA reductase
FLNB: Filamin-B isoform X1
DDT: D-dopachrome decarboxylase
ATP6V1E1: V-type proton ATPase subunit E 1
CHDH: Choline dehydrogenase mitochondrial isoform X1
DDX24: ATP-dependent RNA helicase DDX24
PSMD9: 26S proteasome non-ATPase regulatory subunit 9
NDUFS3: NADH dehydrogenase [ubiquinone] iron-sulfur protein 3 mitochondrial isoform X2
AGXT2: Alanine--glyoxylate aminotransferase 2 mitochondrial
OAT: Ornithine aminotransferase mitochondrial
MT1: Metallothionein
RPL19: 60S ribosomal protein L19
NPC2: Epididymal secretory protein E1 precursor
SLC27A4: Long-chain fatty acid transport protein 4
GLRX3: Glutaredoxin-3
CYFIP1: Cytoplasmic FMR1-interacting protein 1 isoform X2
LOC107051219: Diamine acetyltransferase 2-like
CD36: Platelet glycoprotein 4
RAB10: Ras-related protein Rab-10
ISCU: Iron-sulfur cluster assembly enzyme ISCU mitochondrial
NPM1: Nucleophosmin
RPS11: 40S ribosomal protein S11
UBXN4: UBX domain-containing protein 4 isoform X4
LOC415662: C-factor-like isoform X2
SLC38A4: Sodium-coupled neutral amino acid transporter 4 isoform X1
GALE: UDP-glucose 4-epimerase
PDIA4: Protein disulfide-isomerase A4
EPB41L2: Band 4.1-like protein 2 isoform X4
NDUFB1: NADH dehydrogenase [ubiquinone] 1 beta subcomplex subunit 1
GNLY: Granulysin precursor
DNPEP: Aspartyl aminopeptidase
GIMAP5: GTPase IMAP family member 5
RGN: Regucalcin isoform X1
ACLY: ATP-citrate synthase isoform X3
GSTA4L: Glutathione S-transferase alpha 4 isoform X1
FDX1: Adrenodoxin mitochondrial
SDC4: Protein syndesmos precursor
IFI6: Interferon alpha-inducible protein 6
RMDN1: Regulator of microtubule dynamics protein 1
PSMB7: Proteasome subunit beta type-7
TTC38L: tetratricopeptide repeat protein 38-like
GSTT1L: GLUTATHIONE S-transferase theta-1-like
SLC25A4: ADP/ATP translocase 1
MX: Interferon-induced GTP-binding protein Mx
CYP2C23b: Cytochrome P450 2H2 precursor
GART: Trifunctional purine biosynthetic protein adenosine-3 isoform X1
